# A Qualitative Evaluation of the Motivations, Experiences, and Impact of a Mental Wellbeing Peer Support Group for Black University Students in England and Wales: The Case of Black Students Talk

**DOI:** 10.1177/21582440231218080

**Published:** 2023-12-20

**Authors:** Nkasi Stoll, Anna-Theresa Jieman, Yannick Yalipende, Nicola C. Byrom, Heidi Lempp, Stephani L. Hatch

**Affiliations:** 1 King’s College London, UK; 2 Queen Mary University of London, UK; 3 Black People Talk CIC, London, UK

**Keywords:** social psychology, education, race, ethnicity, qualitative research, mental wellbeing, peer support

## Abstract

Online peer support programs could address mental wellbeing concerns reported by Black students. The current evaluation explored Black university students’ motivations, experiences, and perceived impacts of an online mental wellbeing peer support group (Black Students Talk [BST]) in England and Wales. We conducted two focus groups with Black Students Talk attendees and one with facilitators. Data were analyzed using inductive thematic analysis. Three main themes and eight sub-themes where identified related to (i) Motivation: Impact of racism on mental wellbeing; (ii) Experience: The Black Students Talk experience; and (iii) Impact: Mental wellbeing outcomes. Benefits of Black Students Talk for Black students included advice, rest, validation, and support in the context of their race and experiences of racism. Facilitators had a unique sub-theme concerning their training and support. While racism exists at universities, online peer support can offer valuable benefits for Black students’ mental wellbeing, social connectedness, and Black-only networks. Programs need to be co-created and delivered by trained Black students who receive reflective practice with a Black practitioner. Further independent evaluations using insights from survey and interview data are needed.

## Plain Language Summary

Exploring Black Students Talk, a Mental Health Peer Support Intervention for Black University Students

Researchers studied an online peer support group called Black Students Talk (BST) in England and Wales to see how it helped Black university students with their mental wellbeing. They talked to people who attended or helped run Black Students Talk and asked them why they joined, their opinions about the program, and what they gained from being part of the group. The researchers examined the information using a method called thematic analysis, which helps find important patterns and themes. The study found that racism negatively impacts on the mental health of Black students but Black Students Talk can be a helpful space for Black students to get advice, rest, validation, and support each other. The people who helped run Black Students Talk spoke highly of the much-needed training and support from Black professionals they received. Overall, despite one of the researchers also being one of the program designers, the study showed that online support groups like Black Students Talk can help Black students with their mental well-being and build social connections. To improve the study, the researchers would like to continue evaluating peer support for Black students using surveys and interviews.

## Background

At universities in the United Kingdom (UK), students are exposed to a variety of experiences that make higher education a high-risk period for the onset of mental health problems, including (but not limited to): separation from family, financial problems, sleep disruption, balancing conflicting demands of studies with personal, social and family commitments, and risky behaviors such as recreational drug use and excessive drinking (Cooke et al., 2006; Friedlander et al., 2007; Stallman, 2008).

Black British and international students are exposed to compounding racialized personal, institutional, and systemic challenges at university that may impact their mental health and wellbeing (Stoll, Yalipende, Byrom, et al., 2022). These challenges include anti-Black racism and discrimination from university peers and staff, who may deny their privileges and power over Black university students (Bowden & Buie, 2021; Osbourne et al., 2023; Pryce-Miller et al., 2023). For example, Black students report experiencing or witnessing acts of racism in their university accommodation but reporting and disciplinary procedures fail to keep Black student feeling safe (Cartwright et al., 2022). These experiences can harm Black students’ identity and feelings of belonging at university, further impacting mental wellbeing (Arday, 2018; Osbourne et al., 2021). It is important to acknowledge that Black university students do not only navigate racism in educational institutions. Black people in the UK general population have reported experiences of racial discrimination, abuse or harassment (e.g., being physical attacked, or called names or insulted) in various settings including at work, health care services, public spaces, or in public buildings (Hackett et al., 2020; Wallace et al., 2016). Repeated exposure to racism can impact negatively impact Black people’s mental and physical health, as well as educational and work experiences and outcomes (Hackett et al., 2020; Woodhead et al., 2022).

Black students also face barriers in accessing appropriate support for their mental health. Barriers include cultural naivety and insensitivity of healthcare professionals, and a lack of access to culturally or racially appropriate services (Arday, 2018; Sancho & Larkin, 2020; Stoll, Yalipende, Byrom, et al., 2022). Mental health difficulties compounded by racism and inadequate support, can lead to a perpetuating cycle of health disparities and inequities. Consequently, compared to white students with a mental health condition, Black students with a mental health condition in the UK are less likely to complete their course, earn a first- or upper-second-class (≥60%) degree, and continue their education (Universities UK, 2019).

Peer support can be an effective way for Black university students to cope with the psychological impact of racism, especially when there is a lack of Black mental health practitioners or culturally-responsive services or interventions (Sancho & Larkin, 2020; Thomas & Brausch, 2022). Peer support in mental health care involves people with lived experiences giving and receiving help, based on mutual agreement of what is helpful (Mead et al., 2001). Peer support for university students has been shown to improve mental wellbeing, quality of life, and belonging (Batchelor et al., 2020; Byrom, 2018; Davies et al., 2017). Studies of online peer support groups find many positive aspects that resemble offline groups, including social connectedness and help with problem-solving (Naslund et al., 2016; Shalaby & Agyapong, 2020; Smith-Merry et al., 2019). The option for anonymity offered online is suggested to be important for an open and non-judgmental space (Webb et al., 2008) and participants may find it easier to express themselves online compared to offline (Bargh et al., 2002). For marginalized people, such as racial and ethnic minorities, support groups that are moderated by peers can improve social support and promote health literacy (Harris et al., 2015; Helling & Chandler, 2021).

Black Students Talk (BST) is a mental wellbeing peer support group run by and for Black university students, where students come together to listen, learn, and support their own and each other’s mental wellbeing (www.Blackpeopletalk.co.uk). Group discussion topics, identified from consultation workshops with Black university students and Black Student Wellbeing Study findings (Stoll, Yalipende, Arday, et al., 2022), include race-based trauma, individual and collective grief, neurodiversity, racial and cultural identity, religion, and spirituality. Written Black Students Talk psychoeducational materials were designed and created by the Black Students Talk management team and paid Black student content creators. Training was co-developed with Black students in August 2020, and covered active listening skills, mental health awareness, narrative techniques, problem-solving, safe practices, and confidentiality. In February 2021, the Black Students Talk team was funded by Student Space and the UK Office for Students to deliver online peer support for and by Black university students across England and Wales. From April 2021 to early August 2021 weekly online drop-in sessions were held on Zoom. Sessions were facilitated by paid Black students, who received a day of training from two experienced Black Students Talk facilitators. Facilitators received reflective practice supervision with a Black Counseling Psychologist who specializes in trauma interventions for racialized minorities.

The authors drew on relevant concepts from Critical Race Theory (CRT), to provide a methodological self-consciousness to interrogate the process of delivering Black Students Talk. CRT was applied to explore and challenge racial inequality based on the understanding that racism is embedded as normal practice within society and institutions, rooted in slavery and colonialism (Bonilla-Silva, 1997; Crenshaw, 1991; Crenshaw et al., 1996; Hooks, 1992). CRT in the educational context (CRT-E) argues that institutional norms, privileges, resource allocations, and hierarchies are racialized but mascarade as normative and neutral to benefit white students and staff and disadvantage racially minoritized students (Nguemeni Tiako et al., 2022; Ray, 2019; Smith, 2019; Stewart et al., 2021). For example, education staff sometimes maintain white supremacist curricula and pedagogy to frame ethnic and racially minoritized people within a deficit narrative (Leonardo, 2002; Rollock et al., 2014; Taylor et al., 2016).CRT-E research highlights how racial inequality within UK universities has a detrimental impact on education, health, and socio-economic outcomes of Black and other racialized minority students (Parker & Gillborn, 2020).

The aim of this study was to understand Black university students’ motivations and experiences of attending or facilitating an online drop-in mental wellbeing peer support group in England and Wales. We also sought to understand participants’ perception of the impact of engaging with Black Students Talk. The current study was led by NS as part of the Black Student Wellbeing Study (Stoll, Yalipende, Arday, et al., 2022), who also co-created the grassroots program, Black Students Talk with YY. An independent evaluation could not be conducted due to lack of funding; however, independent researchers were involved in every stage of the data collection, analysis, and write up.

## Methods

### Design and Setting

Focus groups explored Black university students’ motivations, experiences, and outcomes impact of attending or facilitating Black Students Talk. Focus groups provided an opportunity to discuss participants’ perception of the impact of engaging with peer support. Following consultation with Black university students, focus groups were chosen as the most appropriate data collection method because participants preferred collective discussions of shared and unique experiences of marginalization (Rodriguez et al., 2011).

### Sampling and Recruitment

Purposive sampling was employed to invite any Black university student aged 18 or over who either attended or facilitated at least three Black Students Talk sessions to take part in the current study (Shorten & Moorley, 2014). Attendees and facilitators were invited via an email sent from the Black Students Talk project manager, using the program account. All participants had to be a current Black (i.e., African, Caribbean, mixed with Black heritage) British or Black international status undergraduate or postgraduate student at a university in England or Wales. Participants needed to have access to an internet connection to take part, as the focus groups were held online via Microsoft Teams due to the coronavirus pandemic.

### Focus Group Topic Guide

The focus group topic guide was developed in collaboration with four Black university students (Supplemental Appendix 1) using a co-production research approach (Beresford, 2013; Kidd et al., 2017). The questions were about the attendees and facilitators’ motivations, experiences, and the perceived impact of Black Students Talk. Open-ended question stimulated to stimulate group dialogue (O.Nyumba et al., 2018).

### Participants

Thirty Black university students attended Black Students Talk and were invited to participate in the study. Of these, nine attendees (six females, one male, and two non-binary students; five Caribbean and four African students) participated. Eight Black university students facilitated Black Students Talk were also invited to participate in the study. Of these, six facilitators (four females and two male students; one mixed Caribbean and white, two Caribbean, and three African students) participated. Participant demographics have not been reported to ensure that no student is identifiable and at risk of harm due to expressing controversial or negative opinions of their university.

### Procedure

To obtain multiple perspectives, three focus groups were held in late August 2021; two for attendees and one for facilitators. The focus groups were conducted by NS alongside three Black university students who had first-hand experience of poor mental wellbeing at university, and experience conducting focus groups. Each focus group lasted for approximately 2 hr. Discussions were audio-recorded and transcribed verbatim by NS. During the focus groups, participants were given the opportunity to elaborate on subjects they considered relevant and important. Prompts were used to elicit examples, detail and encourage openness (O.Nyumba et al., 2018). All participants were reimbursed with a £10 (love-to-shop) voucher (Ellard-Gray et al., 2015).

### Data Analysis

Qualitative data about participants’ experiences were inductively analyzed (Braun & Clarke, 2006, 2019) supported by NVivo software version 11. Following data familiarization, transcripts were descriptively coded by authors NS and ATJ. The initial coding framework was refined through iterative discussion and coding rounds between both researchers. Themes reflecting relevant patterns within and across interviews were systematically identified and entered into NVivo nodes (codes). Using research memos and discussions, related codes were collated into preliminary themes, gathering all data relevant to each potential theme. These were continually reviewed and adjusted, looking for patterns, and similarities and differences within and across coded extracts. Themes were defined, described, and labeled. Relevant quotes were selected with the assistance of the remaining authors.

### Data Validation

Participants were invited to comment and reflect on draft and final written and oral summaries of the data during two online group workshops led by NS in November 2021 and March 2022. This was crucial in identifying, discussing, and resolving any differences in coding and interpretations and helped facilitate multiple perspectives when interpreting the data (Birt et al., 2016). Participants asked authors to maintain their anonymity by labeling quotes with their role in Black Students Talk and pseudonym, and to remove their ethnicity, gender, and age.

### Reflexivity

During all phases of data collection, analysis, and write up NS kept a self-reflective log on the study and analytic processes, as well as their positionality (Braun & Clarke, 2019). NS used this log to predict, prepare for, and reduce potential challenges. As a Black Caribbean university student with a mental health condition, NS attempted to reduce confirmation bias (McSweeney, 2021) by revealing her pre-existing beliefs and experiences of racism and ableism by drawing and writing in a notebook. During data collection and analysis, she reflected on and challenged how these beliefs and experiences impacted her interpretation of information shared by participants, with the assistance of the other authors.

## Results

**Figure 1.**
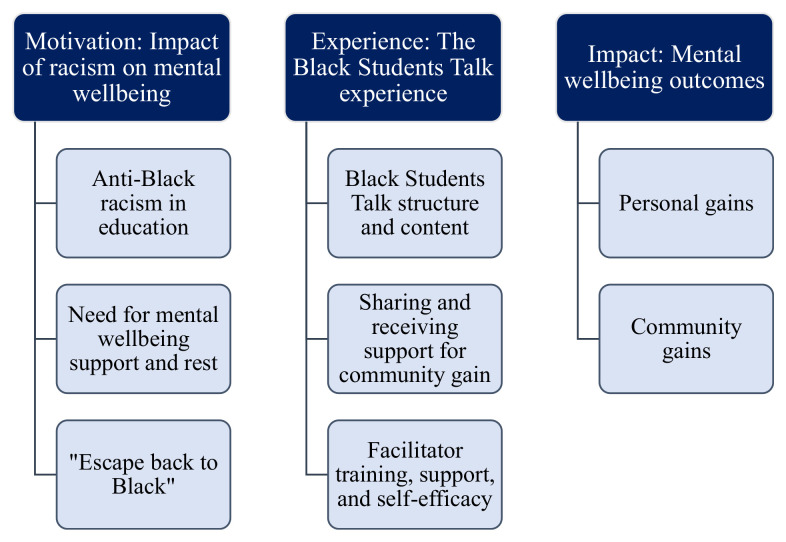
Analytical framework illustrating key themes and sub-themes.

Three main themes emerged: (i) Motivation: Impact of racism on mental wellbeing; (ii) Experience: The Black Students Talk experience; and (iii) Impact: Mental wellbeing outcomes (Figure 1). Eight associated subthemes describe Black students’ motivations, experiences, and impacts from Black Students Talk. We used the identifier “participants” when referring to both attendees and facilitators.

### Theme 1: Motivation: Impact of Racism on Mental Wellbeing

This theme contains three sub-themes: (i) Anti-Black racism in education; (ii) Need for mental wellbeing support and rest; and (iii) “Escape back to Black.” It reflects a theme which emerged across the participants accounts of the impact racial discrimination had on their mental health and educational experiences as they progressed through education.

#### Sub-Theme 1.1: Anti-Black Racism in Education

Several attendees spoke in detail about childhood experiences of racial discrimination during secondary school education.

Because I was a well-behaved smart kid the teachers put me next to the naughty white kid who wasn’t smart because they would use me to help them become more smart or make them behave. I had a teacher call me the ‘N word’ in school when I was eleven (BST attendee, Daniel).

I had a time where I got 100% on an exam and they took off 25% because they said I must have cheated off the white kid next to me, I didn’t obviously (BST attendee, Kofi).

Whilst recalling their experiences of racism, the students appeared visibly distressed (e.g., repeated sighs, looked up toward the ceiling). Other focus group members shook their heads and kissed their teeth in a seeming display of agreement and frustration. This shared experience of distress was echoed in participants’ recollections of racial discrimination from school teachers during a critical developmental stage. Participants reported that racial discrimination had a lasting detrimental impact on their mental health, self-esteem, and identity.

There’s so many moments in my education experience where, as an intelligent young Black girl, white [teachers] have put barriers in my way, have tried to break me down through school, university, postgrad. It really affects your psyche when you’re young, it affects how you feel when you’re already trying to understand where you are in this world. It’s awful (BST attendee, Tessa).

Racial discrimination, prejudice, and harassment from non-Black peers and staff continued at university. In one focus group, participants discussed their reflections of the impact institutional racism has on their mental health, identity, and educational achievements.

You’ve got all these systems that operate perpetually to keep reinforcing the power dynamics and keep you down as Black students…Universities don’t even consider the struggles Black students go through and the differences between different Black students (BST facilitator, Ana).

To cope with racism at university, attendees discussed having to code-switch (e.g., change their behaviors and voice) or hide their anger and frustrations to be perceived by their white peers as socially acceptable. This was experienced as distressing and tiring.

[Non-Black peers and university staff] have all of these perceptions of you. You’re constantly having to think about how you’re perceived in [university] environments and code switch and you have to change how you talk and what you wear or the experiences that you have in your home and family and community life and it’s tiring…I really push back against [racism] and I try to maintain my Blackness in spaces that I go to, but then that has consequences, because you get people trying to dab or make assumptions about what music you listen to, it’s hard to stay quiet and not be affected by [racism] (BST attendee, Lori).

#### Sub-Theme 1.2: Need for Mental Health and Wellbeing Support and Rest

Several participants said they felt lonely and isolated at university because they were the only Black students on their courses, and their academic workload left them with little opportunity to socialize with Black students on other courses. Black Lives Matter (BLM) protests during the coronavirus pandemic were said to have left them further isolated from peers.

You don’t see a lot of other Black students and you don’t really have the time to get to know other [Black] students outside of people that are around you (BST facilitator, Tene).

I needed to find a place where it was comfortable to talk about the hidden or invisible [racism] that we go through in university. It was a very dark time because [my mental health got worse during] the pandemic and BLM it was very isolating…I needed to connect with people during a very difficult time (BST attendee, Kione).

Some attendees said Black people are unable to prioritize self-care and self-compassion because they have internalized the socially conditioned belief that Black people’s worth is grounded in their ability to be productive and successful. Attendees said when they disclosed psychological distress and their need for rest to non-Black peers and university staff they were gaslit by being called “snowflakes” and met with “eye-rolls.” They wanted a space where they could “break down” and “share their suffering” with others.

Being African, it’s just not an option to not go to university. It’s embedded in African kids from a really young age…my friends that hated their degrees, they still had to [complete their university course] because of their parents. You have to finish your degree whether you like it or not, it’s stressful (BST attendee, Banji).Despite discussions about how racial discrimination and prejudice impact their own and others’ mental health and wellbeing, attendees spoke of their desire to focus on the positives and strengths within the Black student community.

To be able to get through all of that [racism] and still be successful at university, we do have superpowers! (BST attendee, Narissa).

Sometimes I look at myself, and my friends, and the [other BST attendees] and I just think wow, we do not give ourselves enough flowers because we achieve so much despite all the [racism at university] (BST attendee, Toni).

#### Sub-Theme 1.3: “Escape, Back to Black.”

Attendees talked about needing mental health support from people of their own race, who have similar ethnicities, cultural backgrounds, beliefs, and life experiences. According to these attendees, Black students are exposed to unique mental health stressors that non-Black students are not exposed to.

Every Black person goes through the same struggle and then not having that understanding from the other racially minoritised people and people who hold power is really tough, and sometimes you just need that escape, back to Black (BST attendee, Narissa).

Similarly to Narissa, other participants recounted that many non-Black students display anti-Black prejudices and discrimination and can be unsupportive of their attempts to advocate for universities to tackle racial inequality. The students claimed that the word “Black” in Black Students Talk was the reason they signed up to the program, to avoid harm from non-Black peers.

Facilitators expressed their desire to challenge mental health stigma among the Black university student community. However, some discussed their anxieties and fears that Black Students Talk would be tokenistic and ingenuine about their commitment to improve the mental health of Black students. Their fears came from attending anti-racist support groups at their universities that they believed were more interested in the observation and study of Black students’ distress as opposed to alleviating it.

Coming into BST, I was like is this gonna be another situation where it seems like they’re doing [BST] for us, but there’s like a white person somewhere with a notebook listening in like ‘so this is how the Blacks are feeling? (BST facilitator, Chima).

### Theme 2: Experience: The Black Students Talk Experience

The theme “Experience: The Black Students Talk experience” contained three sub-themes: (i) “Black Students Talk structure and content”; (ii) “Sharing and receiving support for community gain”; and (iii) “Facilitator training, support, and self-efficacy.” It describes a theme which emerged about the Black students’ participants’ perceptions of the delivery of the Black Students Talk program.

#### Sub-Theme 2.1: Black Students Talk Structure and Content

Despite some initial skepticism, participants fed back that Black Students Talk sessions were well-structured with clear and consistent expectations. They particularly praised the introduction and ice-breaker activities as a way to feel more “confident” and “comfortable” in the space and make friends. Black Students Talk psychoeducational materials were discussed as validating, affirming, and useful discussion tools, especially content pertaining to the impact systemic and institutional racism can have on Black students’ mental health.

Doing these groups, learning narrative therapy techniques, have allowed me to reflect on my behaviours…I’ve learnt it’s fine to encourage people [in my life] to find solutions for themselves (BST facilitator, Ana).

However, the following adjustments to the Black Students Talk structure came up in the focus group conversations. (i) Lecture-style sessions about mental health, wellbeing, and illnesses delivered by a Black expert, were suggested. These would allow attendees to listen, learn, and ask questions. This could run alongside the current session style, which involves psychoeducation and small group discussions. (ii) It was suggested that sessions could run monthly instead of weekly. (iii) More extensive facilitator training on the content of the sessions was recommended.

#### Sub-Theme 2.2: Sharing and Receiving Support for Community Gain

All participants spoke about Black Students Talk sessions being reciprocal in the exchange of advice, support, guidance, as well as personal stories of poor mental health. This was said to be “liberating.”

Lot of times people were very open about their mental health struggles and talked about issues and then somebody else would come in and be like, ‘oh I’ve experienced that before, this is how I cope, these are some healthy coping mechanisms that I’ve implemented’ and I’ve been there taking notes in the background, like that’s actually a really good idea (BST facilitator, Chima).

This sense of community was positioned as a contrast to the racism experienced at university. According to participants, they felt safe to “co-create a communal space for change,” rest, give, and receive comfort, along with celebrating the perceived strengths of the Black student community at Black Students Talk sessions.

All people see is the colour of our skin and other races do not [experience anti-Black racism]. So, it was really nice [BST] was run by people that had actual Black skin…We need to look after ourselves and if we want to rest, we rest, especially the ones who are going through the pipeline to postgrad and beyond. BST is that space where we can rest and be safe (BST attendee, Kione).

The active involvement of Black men sharing their mental health difficulties, talking about their thoughts and feelings, and giving and receiving mental health support during Black Students Talk sessions was praised by female-identifying attendees.

I agree [with another participant], I really liked the involvement of Black men, normally it’s just women. We need Black men and women to see actually not all Black men are feckless or don’t have intellectual rigor, thoughtfulness and emotional challenges. It was so good to see that (BST attendee, Narissa).

Despite sexism not being a Black Students Talk session topic, participants valued the opportunity to discuss how “the patriarchy and racism, and those systems of oppression interact with each other to affect Black men and women.” These responses speak to Black students’ resilience and community-building attempts to counter the negative impacts of racialized gender stereotypes and discrimination.

#### Sub-Theme 2.3: Facilitator Training, Support, and Self-Efficacy

Facilitators unanimously described reflective practice as a useful space for facilitators to listen, learn, and share their thoughts, feelings, and experiences about their personal and work lives. They discussed feeling “metaphorically held” by the Black Students Talk Counseling Psychologist during reflective practice, whom they felt was supportive, competent, and, importantly, because they were Black, could empathize with their life experiences. Some facilitators discussed their desire for reflective practice to be more flexible on the dates and times they were held; others wanted them to be more frequently than fortnightly.

Reflective practice spaces were really good to talk about whatever comes up for you in the group, whatever it was, [work or personal life], she really helped (BST facilitator, Jaden).

Facilitators discussed that Black employees are often not respected or valued for their ideas and contributions at work. They appreciated that the Black Students Talk team listened to and implemented their thoughts, feelings, and opinions about the running and delivery of peer support. All described feeling valued, trusted, and respected.

As facilitators our opinions about how BST runs are really valued and we are really given an opportunity to be involved even in the reforming process, which shows that they value us as employees and that doesn’t happen often at work, as Black people (BST facilitator, Ana).

Facilitators also expressed relief and pride that the attendees received their work well, especially as some were concerned that their perceived privileges (i.e., skin color, class, and health) may affect their relationships with attendees.

I have some privileges…and my worry was because I might be seen as white or really light, they might find it difficult to open up and that would impact how they found the group (BST facilitator, Ana).

Reflective practice and experience in the role were reported to help build their confidence to disclose their thoughts and feelings about their privileges and disadvantages and share their mental health and life experiences with attendees.

Over the months I came to view my role in a more holistic way. I realised that the space was for me as well and it became a space that I looked forward to going to chill, to decompress, to speak about my week, as much as provide that space for other people (BST facilitator, Lori).

### Theme 3: Impact: Mental Wellbeing Outcomes

The final theme “Impact: Mental wellbeing outcomes,” contained two sub-themes: (i) “Personal gains”; and (ii) “Community gains.” This theme emerged during discussions about the observed benefits of participating in the Black Students Talk program during the facilitator and attendee focus groups.

#### Sub-Theme 3.1: Personal Gains

Most participants reported improvements in self-worth, self-esteem, and confidence after attending or facilitating Black Students Talk. These participants discussed feeling better able to describe complex thoughts and feelings amongst themselves and to others, which not only improved their ability to ask for help, but also normalized their mental health difficulties and made them feel less alone and “weird.”

That liberating feeling of having the terminology and the knowledge to validate your experiences [of racial trauma] was invaluable (BST attendee, Kofi).

[BST] was validation because I felt like ‘oh wow, I wasn’t crazy, [racial trauma] is a real issue’. I feel like I’m much more comfortable now supporting myself (BST facilitator, Ana).

While participants above identified having terminology to discuss their experiences, few used the term racial trauma during the focus group discussions. This is a phrase we have added to capture the range of experiences discussed by participants including depression, anxiety, stress, hypervigilance, and low self-worth.

The resources shared and conversations between peers were reported to assist attendees and facilitators in their self-discovery journeys around personal identity and mental health care. One session on Neurodiversity was characterized by attendees as a “breakthrough moment” because it helped them receive a diagnosis for mental health problems, learning difficulties and/or developmental conditions.

It was the techniques that were used to facilitate the sessions. [The session on body image] was just really eye-opening and I really took a lot of techniques from that and applied them to my life (BST attendee, Jaden).

Normally when I’m facilitating [white majority groups], I’ll be like, ‘I’ve learned so much about other people and how other people are different and blah blah blah’, whereas this time it was very, very close to home. It forced me to reflect on a lot of stuff and heal (BST facilitator, Jaden).

Participants used words/phrases like “pleasantly surprised,”“eye-opening,”“life-changing,” and being “forced [to reflect]” to describe the impact the Black Students Talkcontent and discussions had on their understanding of mental health. For many, the sessions were the first time they had been directly asked about or encouraged to think or talk about racism in the context of their mental health. Indeed, a few described the conversations and subsequent personal reflections as “stressful.” Regardless, they unanimously agreed that talking about their mental health in the context of their race, culture, ethnicity, and experiences of racism was “empowering,”“life-changing,” and beneficial for their self-awareness and identity.

Similar to what [another participant] was saying, a lot of the sessions helped me be empathetic and compassionate to myself [because] I didn’t know how much I internalised the strong Black woman trope (BST facilitator, Ana).

Facilitators spoke positively about Black Students Talk being valuable for their career choices, aspirations, and development. Continuing professional development trainings and workshops offered by Black Students Talk were said to provide them with transferrable skills, knowledge, and experiences in peer support and specific mental health conditions and disorders. The trainings and workshops were said to be beneficial for their studies and other employment.

Doing BST was one of the big factors in helping me decide that I don’t want to stay in academia….in academia I am being robbed because I’m not talking to [another Black person] all day. BST really helped reaffirm some [aspects] that I thought about myself for a while (BST facilitator, Chima).

Once you commit yourself to Black empowerment and psychology, you’re not just going to do BST because of professional advancement, because then you’re in the wrong space…humanity, empathy, being genuine is needed for the work to be done well (BST facilitator, Tene).

Similarly to Tene, all facilitators agreed the most important takeaway from Black Students Talk was the giving and receiving of mental health and wellbeing support, rather than career development. This speaks to the importance of civic duty among Black students.

#### Sub-Theme 3.2: Community Gains

BST was reported to benefit participants’ mental health during social unrest and global crises, specifically the Black Lives Matters protests and coronavirus pandemic.

[BST] gave me a space to just cry because [the BLM protests] were heavy, it just brought up all these [traumatic] memories. I needed that help, which luckily I found (BST attendee, Banji).

Indeed, social connection seemed to be an important impact of Black Students Talk, as attendees also engaged in a discussion about Black students’ unequal access to social capital (i.e., expertise, connections, resources, information, and opportunities from others), compared to their white and other racially minoritized peers. This lack of access was said to cause them to fear for their future.

A lot of Black students I know are going through financial difficulties. Black people and social capital, because if there’s one thing that I’m aware of is how little I knew people that were in positions of power to tell me about internships and opportunities that would actually help me to progress, and I think that’s probably one of the best takeaways from [BST] is having social capital, knowing people and connecting with people, that networking is good (BST attendee, Daniel).

Networking with other peers during Black Students Talk provided access to social capital. The happiness displayed in the participant’s facial expressions shows the importance peer support can have on collecting and sharing resources to bolster individual and community wellbeing among Black university students.

## Discussion

This evaluation aimed to explore why students might attend or facilitate an online drop-in mental wellbeing peer support group for Black university students (i.e., Black Students Talk), as well as their experiences and perception of the impacts of the peer support program. Their accounts highlight three themes and eight subthemes: (i) Motivation: Impact of racism on mental wellbeing; (ii) Experience: The Black Students Talk experience; and (iii) Impact: Mental wellbeing outcomes (Figure 1).

Firstly, current and historical racism and the need for social connection, especially in times of global crises and social unrest, was described as the main reason Black university students sought mental wellbeing peer support. Participants described being victims of racism (including racial profiling, harassment and slurs, and gaslighting) from non-Black peers and teaching staff throughout their education journey. For most participants, these experiences motivated them to attend or facilitate a mental wellbeing peer support group for Black students. Similar reports of racism at UK universities have been captured in qualitative studies (Osbourne et al., 2021; Stoll, Yalipende, Byrom, et al., 2022). Consequently, participants experienced their university environments as stressful and lonely, which diminished their sense of control and safety, and elicited feelings of psychological distress including anger, anxiety, fear, disappointment, hopelessness, and low mood. “Code-switching” (i.e., changing their voice and appearance in an attempt to be accepted by their majority white peers) and avoidance of social and teaching university environments was described as ways to cope with racism at university. These reported psychosocial stress responses for being a racially oppressed group member in university and society has been conceptualized as “racial battle fatigue” (Smith et al., 2006, 2016). The current study findings align with CRT-E, which posits that within education institutions, white culture is normative and neutral; and beliefs, actions, or behaviors that are non-white are seen as deviations from the norm, and faced with negative consequences for Black students, including social rejection and psychological distress (Gillborn, 2006; Ray, 2019; Salter & Adams, 2013).

Based on the current study findings, mental wellbeing peer support may provide Black students with a supportive space to recognize, discuss the impacts of, and cope with racial trauma. A valuable aspect of Black Students Talk, reported by the participants, was the reciprocity of storytelling and peer support between Black students which elicited feelings of belonging and safety, positive experiences of the program, self-efficacy, and positive mental health outcomes. This is consistent with existing research findings within non-Black UK student peer support programs indicating that social support is important for mental health and wellbeing, quality of life, engagement, and continuation of university studies (Byrom, 2018). Further, altruism and community support among Black students were highlighted, consistent with previous studies noting their vital role in coping strategies when navigating oppressive and untrustworthy institutions (Burholt et al., 2018; Mosley et al., 2021; Stamps et al., 2021).

Without peer networks at their universities, participants reported feeling alienated and exhausted, with limited social capital due to a lack of people in their social groups being in positions of power and influence (Brouwer et al., 2016). Social capital is an asset, embedded in social relationships, that can be leveraged to improve a student’s mental health, self-efficacy, and educational outcomes (Brouwer et al., 2016). According to the study findings, Black Students Talk can act as an institutional change agent to provide access to information, resources, and support for Black students on how to navigate university environments and reduce psychological distress.

The current evaluation findings suggested mental health programs for Black students need to be Black-only and Black-led for improved mental health and career experiences and outcomes. These findings are validated by the government’s racial disparities commission (2021) who recommended that the term Black, Asian, and Minority Ethnic (BAME) be phased out of use because failing to desegregate between races and ethnicities hides disparities between racial groups as well as people within ethnic groups (Commission on Race and Ethnic Disparities, 2021).

### Strengths and Limitations

While the present evaluation highlights important lessons learnt for universities that are designing and running mental health programs for Black students, the findings need to be considered in the context of several strengths and limitations. Focus group facilitators and data analysts were all Black students, which may have caused social desirability bias in participant responses and focus group dynamics; and confirmation bias during data analysis (Bergen & Labonté, 2019; McSweeney, 2021). However, focus groups where all members are the same ethnicity have been reported to create an environment where participants feel comfortable talking about controversial topics (Greenwood et al., 2014). The depth and length of discussions shared suggests the participants felt comfortable disclosing their experiences. Confirmation bias was mitigated by keeping a reflective diary throughout the research process. Another limitation is that the diversity in definitions of what constitutes a peer-support group means comparisons are difficult between the current evaluation findings and those of other interventions. Others are mostly race-neutral or focused more on supporting academic skills. Further, while focus groups are widely used to understand collective experiences of marginalized populations (Rodriguez et al., 2011), a survey approach would provide participant anonymity and might enable challenges and criticisms of Black Students Talk to be more freely articulated (Bonevski et al., 2014). Moreover, interviews could have further captured individual experiences that participants may not have felt comfortable sharing in a group (Bonevski et al., 2014) Future research using survey or interview methodology would provide further insight into how to improve the experiences and impact of Black student peer support. Additionally, future examination of longitudinal changes in mental health and wellbeing, identity, self-efficacy, social capital, career development would determine the long-term impact participating in Black Students Talk might have on individuals. Lastly, depending on funding availability, future evaluations of Black Students Talk needs to be conducted independently of the management team to limit social desirability bias (Bergen & Labonté, 2019). Despite these limitations, the evaluation of Black Students Talk offers an unique insight into how racism along the UK education pipeline and pre-existing mental health difficulties interact to negatively impact Black students’ and influence them to seek community help, and how online peer support can impact their mental wellbeing and access to social support and networks.

### Implications

Investing into Black student mental wellbeing peer support could repair the distrust between students and mental health services and improve access to, and experiences of, student support. In turn this may improve students’ mental wellbeing. For the program, or similar programs to be effective, Black Students Talk needs to be designed and run exclusively by and for Black students to ensure facilitators and attendees feel confident, trusted, safe, and comfortable to support one another and provide respite from anti-Black racism. Running specific sessions for, and hiring facilitators from, marginalized Black student communities (including first-generation, economically disadvantaged, sexual and gender minority groups) can ensure all Black students are represented and supported. Creation of psychoeducational content on Black student mental health could provide the opportunity to share knowledge and advice about common questions and concerns related to mental health difficulties among Black students, coping techniques, and available support. This might also increase awareness among non-Black students and staff who can signpost Black students to appropriate support. Providing reflective practice with a Black mental health professional is vital so facilitators can debrief after emotionally demanding and re-traumatizing sessions. Further, university student services need to consider training and/or hiring Black mental health professionals to support students with racial trauma. In conclusion, while racism, sexism, and classism are ongoing challenges in university settings, peer support can offer valuable benefits for Black students and the wider student community.

## Supplemental Material

sj-docx-1-sgo-10.1177_21582440231218080 – Supplemental material for A Qualitative Evaluation of the Motivations, Experiences, and Impact of a Mental Wellbeing Peer Support Group for Black University Students in England and Wales: The Case of Black Students Talk Supplemental material, sj-docx-1-sgo-10.1177_21582440231218080 for A Qualitative Evaluation of the Motivations, Experiences, and Impact of a Mental Wellbeing Peer Support Group for Black University Students in England and Wales: The Case of Black Students Talk by Nkasi Stoll, Anna-Theresa Jieman, Yannick Yalipende, Nicola C. Byrom, Heidi Lempp and Stephani L. Hatch in SAGE Open
