# Supplementary material for: A Qualitative Evaluation of the Motivations, Experiences, and Impact of a Mental Wellbeing Peer Support Group for Black University Students in England and Wales: The Case of Black Students Talk
Source: Sage Open. 2023 Dec 20;13(4):21582440231218080. doi: 10.1177/21582440231218080 (PMC11407137; doi:10.1177/21582440231218080)
Supplement: sj-docx-1-sgo-10.1177_21582440231218080 – Supplemental material for A Qualitative Evaluation of the Motivations, Experiences, and Impact of a Mental Wellbeing Peer Support Group for Black University Students in England and Wales: The Case of Black Students Talk [file sj-docx-1-sgo-10.1177_21582440231218080.docx]

*Appendix 1: Black Students Talk (BST) focus group topic guides for attendee and facilitator focus groups*

| **BST Attendee Focus Group Topic Guide** | | |
| --- | --- | --- |
| **No.** | **Questions** | **Prompts** |
| 1 | What were your personal reasons for attending Black Students Talk? | Mental health concerns? Wellbeing concerns? |
| 2 | What were your expectations towards BST before attending a session? What are your opinions of BST now? |  |
| 3 | What are your takeaways from attending BST sessions? | Have you started new habits/behaviours/ways of thinking because of coming to BST session(s)? If so, what things have you started doing?  Changes to social, physical, living, educational environments?  Changes to mental health, mental wellbeing, emotional health? |
| 4 | What worked well and what would you like to be different about the BST sessions? | What about structure? Time? Facilitators? advertisement of the sessions? |
| 5 | What are your thoughts on the topics and themes discussed at the BST session(s)? | What about relevance? topics to add? topics to take away? |
| 6 | Please tell us anything you wish for us to know about how you feel about BST? |  |

| **BST Facilitator Focus Group Topic Guide** | | |
| --- | --- | --- |
| **No.** | **Questions** | **Prompts** |
| 1 | What were your reasons for joining Black Students Talk as a facilitator? | Personal reasons? Professional reasons? |
| 2 | What were your expectations towards BST before facilitating a session? What are your opinions of BST now? |  |
| 3 | What are your takeaways from attending BST sessions? | Have you started new habits/behaviours/ways of thinking because of coming to BST session(s)? If so, what things have you started doing?  Changes to social, physical, living, educational environments?  Changes to mental health, mental wellbeing, emotional health? |
| 4 | What have you learnt about yourself personally and/or professionally since becoming a BST facilitator? |  |
| 5 | What worked well and what would you like to be different about BST training? |  |
| 6 | What worked well and what would you like to be different about BST supervision? |  |
| 7 | Please tell us anything you wish for us to know about how you feel about BST? |  |
